# Supplementary material for: Spin polarized semimagnetic exciton-polariton condensate in magnetic field
Source: Sci Rep. 2018 Apr 27;8:6694. doi: 10.1038/s41598-018-25018-2 (PMC5923979; doi:10.1038/s41598-018-25018-2)
Supplement: Supplementary file 1 — Supplementary Information [file 41598_2018_25018_MOESM1_ESM.pdf]

Supplementary Information for Spin polarized semimagnetic exciton-polariton condensate in magnetic field

Mateusz Król[1,*], Rafał Mirek[1], Katarzyna Lekenta[1], Jean-Guy Rousset[1], Daniel Stephan[1], Michał Nawrocki[1], Michał Matuszewski[2], Jacek Szczytko[1], Wojciech Pacuski[1], and Barbara Piętka[1]

[1]Institute of Experimental Physics, Faculty of Physics, University of Warsaw, ul. Pasteura 5, PL-02-093 Warsaw, Poland
[2]Institute of Physics, Polish Academy of Sciences, al. Lotników 32/46, PL-02-668 Warsaw, Poland
[*]mateusz.krol@fuw.edu.pl

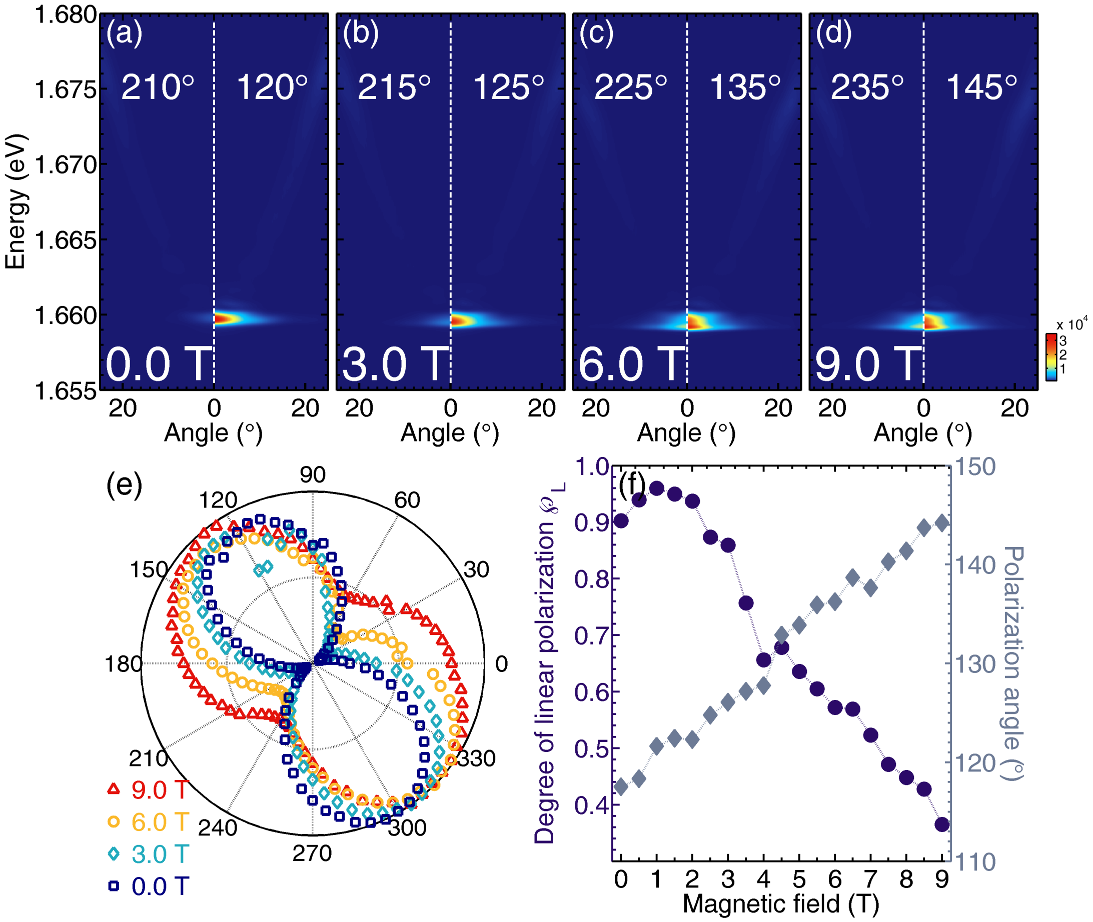

Supplementary Figure 1. Polarization properties of the condensate in external magnetic field. A highly linearly polarized condensate at 0 T, changes to elliptically-polarized condensate after applying external magnetic field. (a–d) Angle resolved PL maps detected in two perpendicular linear polarizations for increasing magnetic field at constant excitation power $P_{ex} = 1.5\ P_{Th}$. (e) Normalized condensate emission intensity dependence on the angle of the linear polarization analyzer. (f) Degree of linear polarization and angle of the linear polarization plane as a function of magnetic field. Lines through experimental points are to guide the eye. The angle of the linear polarization plane rotates by 27° between 0 and 9 T. This effect we assign to the Faraday rotation in the quantum wells, which is additionally enhanced by the multiple reflections inside the cavity.
